# Targetless T cells in cancer immunotherapy

**DOI:** 10.1186/s40425-016-0127-z

**Published:** 2016-04-19

**Authors:** Per thor Straten, Federico Garrido

**Affiliations:** Department of Hematology, Centre for Cancer Immune Therapy (CCIT), Copenhagen University Hospital, Herlev, Denmark; Department of Immunology and Microbiology, University of Copenhagen, Copenhagen, Denmark; Servicio de Analisis Clinicos e Inmunologia, UGC Laboratorio Clinico, Hospital Universitario Virgen de las Nieves, Granada, Spain; Instituto de Investigacion Biosanitaria IBS, Granada, Spain; Departamento de Bioquimica, Biologia Molecular e Inmunologia III, Universidad de Granada, Granada, Spain

**Keywords:** HLA expression, Predictive markers, HLA loss, Immune evasion, Checkpoint inhibition, Adoptive cell transfer (ACT)

## Abstract

Attention has recently focused on new cancer immunotherapy protocols aiming to activate T cell mediated anti-tumor responses. To this end, administration of antibodies that target inhibitory molecules regulating T-cell cytotoxicity has achieved impressive clinical responses, as has adoptive cell transfer (ACT) using expanded tumor infiltrating lymphocytes (TIL) or genetically modified cytotoxic T cells. However, despite clear clinical responses, only a fraction of patients respond to treatment and there is an urgent call for characterization of predictive biomarkers. CD8 positive T cells can infiltrate tumor tissues and destroy HLA class I positive tumor cells expressing the specific antigen. In fact, current progress in the field of cancer immune therapy is based on the capacity of T cells to kill cancer cells that present tumor antigen in the context on an HLA class I molecule. However, it is also well established that cancer cells are often characterized by loss or down regulation of HLA class I molecules, documented in a variety of human tumors. Consequently, immune therapy building on CD8 T cells will be futile in patients harboring HLA class-I negative or deficient cancer cells. It is therefore mandatory to explore if these important molecules for T cell cytotoxicity are expressed by cancer target cells. We have indications that different types of immunotherapy can modify the tumor microenvironment and up-regulate reduced HLA class I expression in cancer cells but only if the associated molecular mechanisms is reversible (soft). However, in case of structural (hard) aberrations causing HLA class I loss, tumor cells will not be able to recover HLA class I expression and as a consequence will escape T-cell lysis and continue to growth. Characterization of the molecular mechanism underlying the lack or downregulation of HLA class I expression, seems to be a crucial step predicting clinical responses to T cell mediated immunotherapy, and possibly aid the selection of strategies that could condition patients for response. Thus, characterization of HLA expression by cancer cells could therefore represent an important predictive marker for immunotherapy of cancer.

## Background

The field of immunotherapy has experienced tremendous breakthroughs over the past few years. To this end, the FDA approval of Sipuleucell T for the treatment of hormone refractory prostate cancer set the stage in 2010 [1], followed by the more recent approvals of the PD-1 and CTLA-4 checkpoint inhibitory monoclonal antibodies (mAb) in melanoma and non-small cell lung cancer (NSCLC) [2]. Importantly, numerous immunoregulatory mAb aimed at blocking inhibitory or boosting stimulatory immune signaling are in development, some of which have already been in clinical testing alone or in combination with the already approved blocking antibodies with promising data, e.g., CD40 agonistic antibody in melanoma [3]. Also, the use of adoptive cell transfer (ACT) using in vitro expanded tumor infiltrating lymphocytes (TIL) have shown very strong clinical efficacy in phase II trials [4, 5], and the administration of T cells harnessed with tumor specific T-cell receptors, show great promise also beyond solid cancer, e.g., myeloma [6]. To the latter point, also soluble monoclonal TCR fusion proteins targeting the HLA/peptide complex and the CD3 molecule are in clinical testing. Despite these tremendous breakthroughs in the field it is clear that only a fraction of patients respond to treatment underscoring the need to characterize predictive biomarkers that would allow selection of patients for individual therapies.

The search for predictive biomarkers is ongoing and current approaches are scrutinizing immune infiltration, expression of the target molecules in the tumor microenvironment, unique genetic hits (e.g., KRAS mutations), phenotypes of T cells among PBMC or TIL, or global mutational load of the cancer cells, just to mention a few. Several of these characteristics of the cancer cells, the microenvironment, or the immune system have shown correlation with response providing interesting new insight into the biology, and in some cases the mechanisms of action in response to therapy. Nonetheless, further work is certainly necessary to elucidate biomarkers that will allow prediction and selection of patients that are prone to respond to treatment. To this end, studies of the actual target of cytotoxic T cells (HLA class I molecules) seem to be strangely missing. At the same time, it has been widely accepted that the majority of tumors lose HLA class I expression. Thus, there is consensus that CD8 T cells are the main effector cells engaged with killing of cancer cells but in most studies it is left unrevealed whether the cancer cells express the actual target: the HLA class I molecules [7].

### Main text

It is well established that the immune system recognize cancer cells, and data are accumulating that spontaneous T-cell responses impact on overall survival [8, 9]. The co-existence of an anti-tumor response with a progressing tumor, highlight that cancer cells eventually escape the immunological response and several mechanisms of immune escape have been suggested [10]. Obviously, characterization of mechanisms of escape could lead to development of immunological strategies that interact with prominent escape routes. To this end, one could argue that the clinical success of targeting the PD-1/PDL-1 axis builds on blocking an interaction that contributes to escape from tumor specific T cells at the tumor site. However, the prerequisite for a successful T-cell response is HLA class I expression on the surface of cancer cells, since clearly the absence or down regulation of HLA class I leave the T cell incapable of recognizing the cancer cell.

HLA class I loss or downregulation has been described in human tumors of different origin with percentages that range from 60 to 90 % [11–14]. Two types of tumor HLA class I alterations are known: 1) caused by reversible or ‘soft’ regulatory defects leading to the coordinated downregulation of genes encoding, HLA class I complex, and components of the antigen processing and presentation machinery; and 2) structural or ‘hard’ irreversible alterations caused by mutational events and chromosomal abnormalities, affecting the HLA class I heavy chain and β2m genes [15]. The reversible ‘soft’ tumor HLA class I deficiencies show low mRNA levels of specific genes (heavy chain, β2m, and APM) that seem to be coordinately down regulated and they can be corrected in vitro by IFN-γ or other cytokines.

Among the ‘hard’ lesions, the loss of heterozygosity (LOH) of chromosome 6p21 is an important mechanism that generates HLA haplotype loss in various human tumors with high incidence. Mutations in β2m gene and loss of another gene copy due to LOH in chromosome 15 are responsable for the irreversible total loss of HLA class I expression, and it has been described in various types of malignancies, both in cell lines and in tumor tissues [16]. It is evident that alteration in the expression of any of the HLA class I molecules can affect both T and NK cell-mediated immunity, with an impact on the tumorigenic phenotype, metastatic capacity, and resistance to immunotherapy in various types of cancer.

The analysis of HLA class I antigens in tumor tissues requires a complex approach since HLA class I heavy chain is highly polimorfic and needs the analysis of the expression of six HLA class I alleles on tumor cells surface which differ among cancer patients [17]. Frozen tissue obtained from cancer patients in coordination with pathologists is commonly analyzed by immunohistology. Microdissection of tumor tissue is currently used to obtain DNA and RNA from particular stroma or tumor areas to define the molecular defects responsible for HLA class I alterations. A more precise definition of the tumor phenotype and of the underlying mechanism of HLA class I defects can be obtained by the combined use of these techniques together with polymerase chain reaction (PCR), comparative genomic hybridization and loss of heterozygosity (LOH) analysis with specific markers spanning the chromosomal region of interest.

High degree of tumor infiltration with T cells is considered to be a good prognostic factor and has been included into a new tumor immunological grading system called “immunoscore” [18]. Different groups including ours have observed in various types of cancers, that the HLA class I negative tumors lack TILs. In contrast, HLA class I positive tumors are characterized by high degree of intratumoral infiltration with CD8+ T cells [19]. In this context, it have been reported that the progression or regression of melanoma metástasis after immunotherapy was associated with HLA class I down or upregulation and T cell low or high infiltration respectively in two mixed responders patients [20], indicating that both parameters are closely related. We favor the idea that the status of intratumoral infiltration, reflects the stage of cancer immune escape during natural cancer progression. At early stages there are more HLA class I -positive tumor cells and many TILs, while at more advanced stages the tumor contains more HLA class I negative escape variants and T cells are outside the tumor tissue restrained in the peritumoral area, in the stroma. T cell immune selection of HLA class I negative variants is a major mechanism to generate tumor escape variants present in many human tumors [21]. The additive effect of studying HLA class I expression in tumors rely in the posibility to define the molecular mechanism responsible for the HLA class I loss or dowregulation. If it is reversible/soft, different immunotherapy approaches will have the capacity to upregulate HLA class I associated antigen presentation and induce tumor rejection [15, 21]. In contrast, if it is irreversible/hard, antigen presentation via HLA I molecules will be bloked and resistant to any type of conventional immunoterapy. The question that come immediately is “what to do when a irreversible/hard mechanism is diagnosed” ? We have suggested and succesful tested “in vitro” and “in vivo” the posibility to transfer wild type HLA class I or beta2 microglobuline genes to restore the HLA class I expression and T cell recognition [22, 23] but other posibilities are open and have been recently discussed [21].

There are recent trials published in which different immune status markers were analyzed including the HLA expression. Tjin et al. [24] studied 27 melanoma tissues before tumor cell vaccination using autologous GM-CSF transduced tumor cells and compared with 16 patients who were not vaccinated. More infiltrating CD4 and CD8 positive cells were found in non progressors compared with progressors and T cell infiltration correlated with overall survival. These authors also reported that the loss of HLA A2 expression in melanoma inversely correlated with the functional activation of melanoma reactive T cell responses indicating that the presence of HLA class I on tumors cells determine the T cell effector function [25]. These results are in agreement with those obtained by Ryschich et al. [8] in pancreatic carcinoma in which T cell infiltration correlated with the HLA class I expression i.e., more T cells in tissues that were HLA class I positive and vice versa suggesting an active ongoing process of T cell immuneselection of HLA class I negative tumor variants. In phase II and III clinical trials using ipilimumab, melanoma patients were typed in for HLA-A2 and HLA-A1 in peripheral blood lymphocytes. Median overall survival was similar in both groups regardless of their HLA class I status [26]. The idea that HLA class I expression can be a predictive marker for the final oucome of a particular immunotherapy trial rely on the tumor tissue analysis for HLA class I expression. We know that the frequency of HLA class I losses in diferent tumor tissues is very high when a carefull analysis is performed including HLA class I ABC total loss, HLA haplotype loss, HLA allelic loss or HLA class I ABC downregulation. These findings suggest that the HLA class I associated tumor escape mechanism is underestimated when performing basic tumor tissue analysis and therefore patients suppossed to be positive in the tumor tissue for a particular HLA class I allele, are not. In this context, the absence of expression in the tumor tissue of three HLA- class I A,B,C alleles (a chromosome six loss) or event a single HLA- class I allele loss can be sufficient to prevent the presentation of the strong tumor rejection antigen to T cells and generate a tumor escape variant [27].

Cancer immunotherapy is finally beginning to deliver on its promise. However, even with the most successful drugs or their combinations, most patients either do not respond or eventually succumb to disease despite initial response. Thus, characterization of biomarkers is essential.

To this end, the tumor microenvironment is clearly of huge importance in the sense that tumors with a (T cell) inflamed environment are more prone to respond to administration of checkpoint inhibitory mAb [28]. Along these lines, response to PD-1 therapy has been shown to correlate with expression of PDL1 in the tumor microenvironment [29]. The immunogenicity of the cancer cells is similarly important. Thus, mutations may lead to immune responses against neo-antigens, which may be broadly applicable e.g., KRAS mutations in pancreatic cancer [30], or may be patient specific unique mutations matching only a single HLA allele [31]. To this end, it is clear that at least a fraction of the antigens recognized by TIL are mutated peptides [32], and through technological advances the possibility to routinely target such peptides is no longer wishful thinking or belong in a distant future [32].

Thus, it is clear that both in terms of strength of the immune responses, i.e. what can be induced by unleash of in situ T cells by blocking of inhibitory molecules, as well as in terms of specificity by targeting of mutated peptides, the tools at hand are much improved.

As mentioned, however, there is an urgent call for predictive markers for response, and there seem to be a misconception in the field. Thus, considering PD-1 therapy as “targeting therapy”, it obviously makes at least some sense to study expression of PDL-1 also underscored by the correlation with response. However, it should be kept in mind that PDL-1 is really not the target that directly leads to killing of cancer cells. Similarly, concerning neo-antigens, the mutated protein and the peptide derived thereof is a pre-requisite but is in fact not the target. The target is the mutated peptide in context of the appropriate HLA molecule. Goes without saying – the same can be said about any immunotherapy that relies on a T-cell effector arm, e.g., therapeutic vaccination.

Hence, over the past few years we have discovered tools to target cancer cells with much more powerful approaches while at the same time with a much higher degree of specificity. However, quite often the target is out of sight, in the sense that although HLA molecules are the target, predictive markers are searched high and low while HLA molecules seem somewhat out of the spotlight.

## Conclusion

We propose that expression of HLA class I – global as well as at the level of individual alleles, depending on the immunological targeting strategy - is studied carefully as a potential predictive biomarker in immunotherapy. Prospectively, hard lesions e.g., genetic loss of both alleles of the β2m in chromosome 15 or an HLA haplotype loss affecting one chromosome six should represent exclusion criteria [33]. In retrospective analyses, HLA expression data can be used to correlate with response to therapy. As given above concerning soft lesions, a low level expression of HLA class molecules may be rectified by interferon-γ (IFN-γ) or others TH1 type cytokines. Potentially, data from retrospective analyses can form the basis of “conditioning” prior to treatment thereby altering the microenvironment. To this end, targeting of antigens expressed not solely by cancer cells but by also cancer stromal cells, e.g., IDO-1 [34] could lead to influx of immune cells which in turn secrete IFN-γ, enabling subsequent targeting also of the true target i.e. the HLA molecules expressed by cancer cells. Even less invasive strategies may be relevant. In conclusion, analyzing the expression of the true target - the HLA molecule - will surely exclude patients that have very limited chance for response, and also identify a group of patients for which conditioning that leads to upregulation of HLA molecules will increase the chance of response to therapy [7].

## Abbreviations

CTLA-4: cytotoxic T-lymphocyte-associated protein 4
HLA: histocompatibility leucocyte antigen
IFN-γ: interferon-γ, β2m, Beta-2-microglobulin
mAb: monoclonal antibodies
NK cell: natural killer cell
PD-1: programmed death-1
TCR: T cell receptor
TIL: tumor infiltrating lymphocytes
